# 

**Published:** 1889-10

**Authors:** 


					﻿OCTOBER, 18BQ.
OLD SERIES
Vol. XXV
<7 1855 <
NEW SERI tv
Vol. VI -NoK
'Olb (JFDURNA^^s

yw. F. WESTMORELAND, M.D.
H. V. M. MILLER, M.D., LL.D.
VIRGIL 0. HARDON, M.D.
F. W. McRAE. M.D.. Man. & Pro.
SUBSCRIPTION t 12.50 A YEAR
				

